# Ileoanal pouch revision and excision surgery in a newly established pouch center: requirements and costs for service provision

**DOI:** 10.1007/s13304-024-01768-9

**Published:** 2024-02-29

**Authors:** Valerio Celentano, Yu Jin Lee, David Rebelo, Triantafyllos Doulias, Sarah Mills, Carlo Alberto Manzo

**Affiliations:** 1 Inflammatory Bowel Disease and Ileoanal Pouch Surgery Centre, Chelsea and Westminster Hospital NHS Foundation Trust, 369 Fulham Road, London, UK; 2 Department of Surgery and Cancer, Imperial College London, London, UK; 3 The Chartered Institute of Management Accountants (CIMA), London, UK; 4 Kettering General Hospital, Northampton, UK

**Keywords:** Ileoanal pouch, Ulcerative colitis, J-pouch, Inflammatory bowel disease, IPAA

## Abstract

Complications of ileoanal pouch surgery affecting function and quality of life may require surgical correction or pouch excision. The management of patients with pouch dysfunction requires a multidisciplinary approach and demand for service provision include multiple healthcare professionals and resources. The aim of this study is to present the service requirements, and surgical outcomes for redo pouch surgery and pouch excision, with cost analysis of the required resources. All patients undergoing surgery for revision or excision of the ileoanal pouch from June 2021 to May 2023 were prospectively included. Patient undergoing only diagnostic procedures, or perineal procedures were excluded. Outcomes within 30 days of surgery were collected, including readmissions and re-operations. Cost analysis of all investigations, outpatient appointments and procedures prior to pouch revision or pouch excision was conducted. Twenty patients were included during the 24 months study period: 13 underwent abdominal revisional pouch surgery, 7 had ileoanal pouch excision. 15 patients (75%) were tertiary referrals from other hospitals in the UK. The median interval between index IPAA surgery and revision was 113 months. Three multidisciplinary clinical appointments, two imaging modalities, and at least one invasive day-surgery procedure were required for each patient prior to surgery. Expertise and infrastructure are needed for indication and peri-operative management of patients with pouch dysfunction requiring pouch revision or pouch excision. We estimated a starting cost of £22.605 ($29.589) for provision of pouch revision or excision surgery for investigations and treatments from referral to the pouch unit to surgery. This likely represents an underestimate as only accounts for procedures performed since referral with pouch dysfunction.

## Introduction

Mechanical complications of the ileoanal pouch affecting function and quality of life may require surgical correction or pouch excision [1]. It is well known that ileoanal pouch surgery is performed infrequently, making it difficult for surgeons and multidisciplinary team members to develop expertise in ileoanal pouch anastomosis (IPAA) surgery [2], and even more in revisional and excisional surgery. The requirement for service provision includes multiple healthcare professionals and resources, with implications for providers on the sustainability of such services [3], particularly in case of small volume of activity. Colorectal surgeons performing revisional pouch surgery will also often require support from other surgical teams, such as plastic surgeons, urologists, and gynecologists. The aim of this study is to present the service requirements, surgical volumes, and outcomes, with cost analysis of the required resources.

## Methods

### Study design

All patients undergoing surgery for revision or excision of the ileoanal pouch from June 2021 to May 2023 were prospectively included. Patient undergoing only diagnostic procedures, or perineal procedures were excluded. Patients having only ileostomy formation with the ileoanal pouch kept in situ were also excluded. Collected data included patient baseline characteristics, history and duration of disease prior to IPAA, and indication for revisional or excisional surgery. Outcomes within 30 days of surgery were collected, including readmissions and re-operations.

All pre-operative investigations, outpatient clinic appointments, and procedures prior to offering a pouch revision or pouch excision were recorded, and an in-depth financial assessment of the costs required for surgery, postoperative stay, and follow-up within 30 days of surgery was conducted, supervised by the finance officer of the Hospital. The study protocol was developed according to the STROBE checklist [4]. Indication for excisional and revisional surgery was discussed at the dedicated IBD MDT, and more recently, also at a multi-hospitals pouch MDT, co-chaired by the corresponding author of this study [5].

### Primary and secondary outcomes

The primary outcome was the financial cost of the entire patient pathway from referral to follow-up after surgery. The secondary outcome was 30-day postoperative morbidity.

### Statistical analysis

Categorical variables are presented as frequency or percentage and were compared with the use of the chi-square test or Fisher’s exact test, as appropriate. Continuous variables are presented as mean (± standard deviation) or median (range) and were compared with the use of Student’s t test. The Mann–Whitney U test was used for continuous, not normally distributed outcomes.

Statistical analysis was performed using the Statistical Package for Social Sciences (SPSS version 16.0; SPSS, Chicago, IL, USA). All reported *p* values were two-tailed, and *p* values of less than 0.05 were considered to indicate statistical significance.

### Ethics

The study is conducted in accordance with the principles of the Declaration of Helsinki and ‘good clinical practice’ guidelines. Informed consent was obtained from the patients. The study was approved by the local audit committee, as part of a service evaluation of the ileoanal pouch service.

## Results

Twenty patients were included during the 24 months study period: 13 underwent abdominal revisional pouch surgery and 7 had ileoanal pouch excision. Fifteen patients (75%) were tertiary referrals from other hospitals in the UK. The median interval between index IPAA surgery and revisional surgery was 113 months (range 12–406). A median of three multidisciplinary clinical appointments (range 2–5), two imaging modalities (range 1–5),and at least one invasive day-surgery procedure was required for each patient prior to surgery. The details of the procedures performed are presented in Table 1.

**Table 1:** Indication for surgery and procedures performed

	Indication for surgery	Interval from Index IPAA formation (months)	Pouch configuration	Procedure performed	Comments
1	Recurrent small bowel obstruction	36	J	Laparoscopic pouchopexy	
2	Recurrent small bowel obstruction, recurrent pouchitis	50	J	Laparoscopic pouchopexy	
3	Recurrent small bowel obstruction, deep infiltrating endometriosis affecting left ureter	12	J	Laparoscopic pouchopexy and ureterolysis	
4	Chronic pelvic sepsis	20	J	Open resection and refashioning of blind end of the pouch	
5	Recurrent small bowel obstruction, weight loss	354	W	Laparoscopic pouchopexy and adhesionlysis	
6	Emergency small bowel obstruction	406	W	Open in situ pouch augmentation with formation of new pouch inlet	
7	Recurrent cuffitis and pouchitis, with long retained rectum	133	J	Open pouch advancement with redo pouch	Excision of long rectal cuff (old pouch excised due to fibrosis)
8	Entero-cutaneous and pouch vaginal fistula	140	J	Pouch excision and hysterectomy	
9	Chronic pouchitis and pouch inlet stricture	436	W	Open strictureplasty of pouch inlet	Heineke-Mikulitz strictureplasty
10	Chronic pouchitis and acute small bowel obstruction	70	J	Open pouch inlet strictureplasty	Jaboulay strictureplasty
11	Pouch inlet stricture, weight loss	302	J	Open redo pouch	Previous pouch excised as fibrotic. Histology: Crohn’s disease
12	Recurrent anastomotic fistula	119	J	Open pouch advancement	Able to re-use existing pouch
13	Pelvic pain and recurrent pouchitis	378	S	Laparoscopic pouchopexy	
14	Recurrent pouchitis and chronic poor function	94	J	Pouch excision	
15	Pouch vaginal fistula and previous high output jejunostomy for peritonitis	126	J	Pouch excision. Refashioning of ileostomy. Cholecystectomy	
16	Pouch vaginal fistula and previous entero-cutaneous fistula	80	J	Pouch excision and flap repair of pouch vaginal fistula	Martius flap to repair vaginal fistula
17	Entero-cutaneous fistula, poor pouch function, multiple incisional hernias	120	J	Pouch excision and abdominal wall reconstruction	
18	Entero-cutaneous fistula, chronic pelvic sepsis, multiple incisional hernias	71	J	Pouch excision and abdominal wall reconstruction	
19	Recurrent pouchitis, suspected NET of the Pouch	107	J	Pouch excision	NET of the pouch inlet
20	Pelvic sepsis and recurrent pouchitis	50	J	Redo pouch	

*NET* neuroendocrine tumour

Patients’ baseline characteristics, pre-operative investigations, and surgical outcomes are presented in Table 2. There were no mortalities and no re-operations within 30 days of surgery. Four patients (20%) developed Clavien–Dindo 3 or higher complications, two of these were in patients who had a pouch excision, and consisted of bile leak treated with ERCP in a patient who had simultaneous cholecystectomy for large gallstone, and upper gastrointestinal bleeding requiring OGD and transfusion, whilst two occurred in patients who underwent redo pouch and consisted in one entero-cutaneous fistula treated conservatively and closed within 90 days of surgery, and one stoma site fistula treated conservatively.

**Table 2:** Postoperative outcomes

Age	49 (21–66)
Procedures (*n* = 20)	Pouch excision: 7 (37%) Redo pouch: 6 (30%) Pouchopexy: 5 (26%) Pouch inlet strictureplasties: 2 (11%)
Approach	Open: 14 (74%) Laparoscopic: 5 (26%)
Investigations prior to surgery	EUA and pouchoscopy: 11 (58%) CT: 8 (42%) MRE: 17 (90%) MRI pelvis: 17 (90%) Diagnostic laparoscopy: 4 (21%)
Multiple specialists involved at surgery	Urologist: 12 (63%) Plastics: 2 (11%) Gynecology: 5 (26%)
Operating time	300 min (66–531)
Length of stay	10 days (3–61)
30-day morbidity	4 (20%)
Re-operations	0
Readmissions (including 30 and 90 days)	4 (20%)
Estimated cost	Total cost: 22.605 - Pre-operative: 3.920 - Surgery and postoperative follow-up: 18.685

*EUA* examination under anesthesia, *MRE* magnetic resonance enterography, *MRI* magnetic resonance imaging, *CT* computer tomography, *ICU* intensive care unit

The estimated costs from the referral to the service till 30 days after surgery were a median of £ of £22.605 ($29.589), see Table 3.

**Table 3:** Average cost of common invasive procedures performed on patients with ileoanal pouch dysfunction

Procedure	Cost range (£)
Examination under anesthetics and pouchoscopy	1.450–1980
Magnetic resonance imaging pelvis and small bowel	430–1120
Ileoanal pouch excision surgery	11.000–19.500
Ileoanal pouch revision surgery	5.400–17.350
Diagnostic laparoscopy	1.800–2.700

## Discussion

Several multi-specialty clinical reviews, imaging modalities, and invasive procedures are required for patients with pouch dysfunction, prior to undergoing revisional or excisional ileoanal pouch surgery. The results of our study report an average of 3 clinical appointments, 2 MDT discussions, and at least 2 imaging modalities and 1 day surgery procedure. This is likely an underestimate, as it only considers the pathway of the patient from the referral at the tertiary center, whilst most of the patients would have already had many investigations at their local hospitals over the previous years. Moreover, patients with an ileoanal pouch often require counselling, investigations, and treatment in primary care, with added costs and service requirements, which have not been captured in our study. Our results highlight the significant resources required for the necessary care to be offered to this selected group of patients, with complex disorders of the pouch resulting in recurrent admissions to hospital, treatments, and reduced quality of life. The need for a multidisciplinary team, ranging from specialist surgeons and gastroenterologists, nutritionists, specialized nurses, pathologists, and radiologists, cannot be over-emphasized. Moreover, other surgical specialties are required to support the operation in more than half of the patients, as in our case series, which involved urologists, plastic surgeons, and gynecologists.

This has obvious financial implications, and our cost evaluation has estimated £22.605 ($29.589) required for the pre-operative evaluation, surgery, and 30-day postoperative period. Again, this likely represents an underestimate, as cannot include all the pathway of the patient pre-referral, the costs of medical treatment, and the implication on quality of life, social functioning, and ability to work. This is a limitation of our study, like other health-economics evaluations. Nevertheless, our cost evaluation, based on prospectively collected data, could guide service providers, wishing to develop a specialized service for patients with pouch dysfunction. Whilst our pathway has generated a significant increase in the volume of clinical and surgical activity [3], its financial sustainability remains to be demonstrated in the long term. All patients undergoing revisional pouch surgery in our center are admitted postoperatively to the high dependency unit, and our results show a postoperative length of stay of 10 days, with significant input from occupational therapist, pain team specialists, and physiotherapists.

Centralizing the care of patients undergoing primary and revisional ileoanal pouch surgery in few dedicated pouch units could result in increased volume of surgical activity, making it more appropriate for those units to invest into the service, with resulting benefits for training and nurturing expertise across different specialties. As a matter of fact, gaining experience in pouch surgery is not easy, as the procedure is performed infrequently across many hospitals, as reported by the UK Pouch registry [1], outlining that the average number of pouches performed in English institutions was just three cases per year and one-quarter of the surgeons undertaking this operation had performed only one case over the last 5 years. Careful patient selection and counselling, appropriate surgical technique [6] and follow-up pathway, can provide optimal functional outcomes and good quality of life in many patients [7]. However, IPAA is a complex procedure, associated with short- and long-term morbidity. Several complications following IPAA surgery can lead to pouch failure, defined as functional failure of the pouch requiring pouch excision or permanent diversion, and reported rates can be up to 20% [8], highlighting the need for ongoing specialized care required for patients after the primary IPAA procedure, as a significant proportion will require ongoing treatments and interventions. The definition and wording of pouch failure does not consider patients quality of life, and a more holistic and comprehensive patient’s assessment tool must be developed. A combination of clinical, endoscopic, and radiological [9] evaluation is essential to appropriately diagnose and address pouch dysfunction, and our study has the merit of highlighting not only the need for this multidisciplinary involvement, but also the financial impact of the infrastructure needed, and expertise required. However, many pouch disorders can be treated medically or endoscopically [10], whilst our study focused on the mechanical and septic complications requiring surgical correction. The main limitation of our study is that the cost analysis we conducted under-estimates the costs of pouch disorders, as cannot include the pre-referral pathway, or the cost for primary care providers. More importantly, the implications on patients’ health, quality of life, and work and social life are obviously much more significant, but we considered them outside of the scope of this manuscript due to the small sample size and short follow-up, having these already been reported extensively [11, 12].

## Conclusions

We estimated a starting cost of £22.605 ($29.589) for provision of ileoanal pouch revision or excision surgery for investigations and treatments from referral to the pouch unit to surgery. Expertise and infrastructure are needed for appropriate indication and peri-operative management of these uncommonly performed surgical procedures.

## Data Availability

Data will be made available on request.
